# A survey study of the psychological impact of COVID-19 pandemic on physician parents

**DOI:** 10.1186/s12245-021-00370-9

**Published:** 2021-08-30

**Authors:** Jean C. Y. Lo, E. Lea Walters, Deena I. Bengiamin, Emma M. Spivey, Katelyyn C. Wegrzyn, Ellen Reibling

**Affiliations:** Department of Emergency Medicine, Loma Linda University Medical Center, 11234 Anderson Street, Loma Linda, CA 92354 USA

To the Editor,

We enjoyed reading the comprehensive review article “Disaster management of the psychological impact of the COVID-19 pandemic.” We especially read with interest the effects on health care workers. The review stated that these effects are more pronounced in acute care physicians for multiple reasons [1]. In addition to the reasons stated by Sheek-Hussein et al., emergency physicians may experience even greater stress. For example, Preti et al. noted that the flood of information regarding the disease lead to increased stress level [2], while Elbay et al. found those who cared for an increased number of COVID-19 patients and those who had increased weekly working hour were factors associated with a higher level of depression, anxiety, and stress [3].

We conducted an online survey of physicians at a tertiary hospital, *n* = 101, that yields similar results. Our study reveals that emergency physicians experienced greater level of stress during the COVID-19 pandemic compared to physicians who do not work directly with COVID-19 patients (*p* = < 0.001, CI − 0.77–2.55).

We also found that physicians with children have higher levels of stress than physicians with no children (*p* = 0.01, CI 0.17–2.57). Several factors were identified. Physician parents concerned about exposing their children to COVID-19 were more stressed (*p* = < 0.001, CI 0.82–3.56). For physician parents, self-isolation was not always an option after work, possibly placing family members at risk of infection. Conversely, physician who self-isolate lost the benefits of family and social contact [4]. Physician parents also had the added stress of comforting fearful children who developed anxiety for their parent’s safety from infection, along with navigating through their children’s personal hardships relating to the social inactivity and restriction the pandemic elicited [5].

Finally, for parents with school-aged children during the pandemic, the switch to homeschooling or remote learning was another new and unexpected challenge. Of the physician parents, those who were required to homeschool their children expressed higher levels of stress than physician parents whose children were in physical school or hybrid learning (*p* = 0.01, CI 0.43–3.26). Physicians with school-aged children, who are already tasked with a demanding career and have created a structured balance between their home life and work life, became tasked with facilitating remote learning. Physicians were coming home after a long day of work during the pandemic to now ensure their children are receiving education. This new task put added stress to their already busy schedules, which included helping their children keep track of their school work and/or virtual learning schedules [6]. Specifically, physician mothers, especially those with elementary school-aged children, found they had increased levels of stress when attempting to manage their children’s remote learning, or new homeschooling needs, when compared to those physicians without children, or a child within this age group [5]. Ultimately, these physicians with school-aged children lacked the ability and time to care for their mental health which led to increased stress level.

In conclusion, our study revealed the physicians who had higher stress level were emergency physicians, physicians with children, and physicians who homeschooled their children.

## Data Availability

The datasets used and/or analyzed during the current study are available from the corresponding author on reasonable request.
